# Supplementary material for: A Likelihood Approach for Real-Time Calibration of Stochastic Compartmental Epidemic Models
Source: PLoS Comput Biol. 2017 Jan 17;13(1):e1005257. doi: 10.1371/journal.pcbi.1005257 (PMC5240920; doi:10.1371/journal.pcbi.1005257)
Supplement: S1 File — (TAR.GZ) [file pcbi.1005257.s014.tar.gz › HSPH_Online-SI-Revision/MSS10/n100-severe/n100-severe_NEW2_table-4.pdf]

1	newly 3 weeks spec	6483.	4770.52	{4190., 5559.}	26.4149	0.135735	newly 3 weeks cumul	16417.	13636.7	{12252., 15001.}	16.9357	0.081977	newly 5 weeks spec	6768.	3559.31	{2881., 4715.}	47.4097	0.287127	newly 5 weeks cumul	30019.	21527.9	{19029., 24896.}	28.2856	0.146679	newly 54 weeks cumul	56585.	30380.6	{25845., 38564.}	46.3098	0.275881
2	newly 3 weeks spec	9252.	10853.8	{9743., 11807.}	17.4228	0.0687159	newly 3 weeks cumul	20576.	23798.4	{21409., 25902.}	15.928	0.0631704	newly 5 weeks spec	7700.	8391.61	{7553., 8955.}	9.83442	0.0379524	newly 5 weeks cumul	37668.	42979.7	{39320., 46385.}	14.1681	0.0567023	newly 61 weeks cumul	50118.	56442.1	{51966., 57005.}	12.6183	0.0478467
3	newly 3 weeks spec	13618.	13824.3	{12880., 14536.}	4.69783	0.0204502	newly 3 weeks cumul	28056.	28396.1	{25823., 30165.}	4.68402	0.0202998	newly 5 weeks spec	10200.	11322.7	{9021., 13397.}	15.4675	0.0629136	newly 5 weeks cumul	51910.	54002.	{49831., 58733.}	6.52907	0.0276989	newly 62 weeks cumul	64948.	73036.7	{58692., 89166.}	15.4391	0.0609164
4	newly 3 weeks spec	8496.	5921.45	{5249., 6507.}	30.3031	0.158368	newly 3 weeks cumul	19445.	15329.6	{13529., 17410.}	21.1642	0.105519	newly 5 weeks spec	8732.	4061.59	{3677., 4435.}	53.4861	0.333582	newly 5 weeks cumul	37352.	24746.3	{22866., 26681.}	33.7485	0.179701	newly 60 weeks cumul	61359.	31584.8	{30392., 32926.}	48.5246	0.288582
5	newly 3 weeks spec	5838.	4327.75	{3758., 4878.}	25.8693	0.132215	newly 3 weeks cumul	15689.	13243.	{11917., 14697.}	15.6085	0.0751973	newly 5 weeks spec	5980.	2903.17	{2345., 3410.}	51.452	0.318388	newly 5 weeks cumul	27938.	19851.4	{17483., 21984.}	28.9447	0.150119	newly 54 weeks cumul	56535.	26634.6	{22772., 30007.}	52.8882	0.329349
6	newly 3 weeks spec	8007.	6255.89	{4723., 8183.}	23.7466	0.123009	newly 3 weeks cumul	17018.	14605.6	{11275., 18266.}	17.4587	0.0868259	newly 5 weeks spec	9118.	5370.39	{4265., 7920.}	41.1032	0.242033	newly 5 weeks cumul	35492.	26343.9	{21122., 35064.}	26.3044	0.138432	newly 60 weeks cumul	63071.	36457.2	{31613., 50244.}	42.1966	0.245175
7	newly 3 weeks spec	9834.	10321.6	{9348., 11196.}	7.72697	0.032753	newly 3 weeks cumul	19745.	20790.7	{19290., 22517.}	7.42461	0.0312713	newly 5 weeks spec	11408.	12119.	{9001., 13022.}	12.8271	0.0553631	newly 5 weeks cumul	43078.	45317.8	{38661., 48429.}	8.69286	0.0370944	newly 61 weeks cumul	66246.	82025.9	{53642., 90740.}	29.1876	0.112213
8	newly 3 weeks spec	6637.	5493.57	{4400., 6445.}	17.662	0.0890636	newly 3 weeks cumul	16097.	13936.5	{11620., 15749.}	13.8425	0.0676934	newly 5 weeks spec	7091.	4862.1	{3738., 6279.}	31.4328	0.175496	newly 5 weeks cumul	30302.	24290.1	{19947., 28742.}	19.8698	0.101096	newly 57 weeks cumul	53290.	37058.1	{29972., 46108.}	30.4595	0.166611
9	newly 3 weeks spec	10162.	7298.19	{6700., 7882.}	28.1816	0.144706	newly 3 weeks cumul	16274.	12468.	{11419., 13425.}	23.3872	0.116711	newly 5 weeks spec	13814.	14171.8	{13395., 14763.}	3.99602	0.0170579	newly 5 weeks cumul	44791.	38358.7	{36300., 40592.}	14.3608	0.0677511	newly 62 weeks cumul	61547.	81892.7	{69770., 90654.}	33.0571	0.12202
10	newly 3 weeks spec	8608.	6516.61	{5203., 8672.}	25.5331	0.133304	newly 3 weeks cumul	19464.	16340.2	{12975., 19944.}	17.8504	0.0897582	newly 5 weeks spec	8668.	4715.51	{3718., 7251.}	45.5986	0.276918	newly 5 weeks cumul	37387.	27101.5	{22781., 36027.}	27.7318	0.14684	newly 55 weeks cumul	57915.	35183.	{30765., 49128.}	39.2506	0.222975
11	newly 3 weeks spec	9885.	11741.9	{8074., 14520.}	28.1607	0.108341	newly 3 weeks cumul	20715.	26416.3	{16567., 35432.}	36.866	0.135526	newly 5 weeks spec	9915.	4294.92	{2533., 6457.}	56.9388	0.396111	newly 5 weeks cumul	41751.	39078.1	{30009., 45762.}	13.2368	0.0634062	newly 61 weeks cumul	59863.	42184.3	{34771., 47370.}	29.5319	0.155698
12	newly 3 weeks spec	6820.	4837.52	{4365., 5205.}	29.0686	0.150185	newly 3 weeks cumul	17074.	13807.8	{12413., 14718.}	19.1434	0.0933713	newly 5 weeks spec	6925.	3394.28	{3027., 3661.}	50.9851	0.31081	newly 5 weeks cumul	31324.	21480.1	{19850., 22830.}	31.4259	0.164536	newly 54 weeks cumul	59414.	28828.3	{27022., 30187.}	51.4789	0.314471
13	newly 3 weeks spec	6696.	4605.21	{4056., 5074.}	31.2245	0.164321	newly 3 weeks cumul	17080.	14014.1	{12126., 15989.}	18.072	0.0888797	newly 5 weeks spec	6771.	2736.26	{2355., 3125.}	59.5885	0.396499	newly 5 weeks cumul	30784.	20491.8	{18436., 22684.}	33.4336	0.17807	newly 54 weeks cumul	57756.	25459.4	{23867., 27343.}	55.919	0.356627
14	newly 3 weeks spec	5021.	3859.87	{3332., 4391.}	23.1255	0.116558	newly 3 weeks cumul	12730.	10885.1	{9155., 12212.}	14.9353	0.0723764	newly 5 weeks spec	5391.	3024.1	{2743., 3328.}	43.9047	0.252249	newly 5 weeks cumul	23614.	17500.2	{15398., 19215.}	25.8905	0.131592	newly 54 weeks cumul	54929.	25421.8	{23772., 26738.}	53.7188	0.334988
15	newly 3 weeks spec	6167.	4410.23	{3603., 5154.}	28.5236	0.149673	newly 3 weeks cumul	14161.	11027.4	{8978., 12916.}	22.7153	0.115433	newly 5 weeks spec	6966.	4150.21	{3650., 4555.}	40.4219	0.226485	newly 5 weeks cumul	28290.	19740.	{17215., 22169.}	30.2227	0.158718	newly 56 weeks cumul	48068.	30958.5	{28914., 33142.}	35.5944	0.191682
16	newly 3 weeks spec	5400.	4446.33	{3729., 4996.}	17.662	0.0869395	newly 3 weeks cumul	13040.	11561.9	{9670., 13060.}	11.918	0.0575179	newly 5 weeks spec	6295.	3901.47	{3459., 4358.}	38.0227	0.209506	newly 5 weeks cumul	25735.	19877.3	{17205., 22078.}	22.7615	0.114005	newly 55 weeks cumul	58209.	30086.7	{27750., 32264.}	48.3126	0.287466
17	newly 3 weeks spec	7444.	4642.06	{3413., 5745.}	37.6402	0.214558	newly 3 weeks cumul	14892.	10109.3	{7160., 13091.}	32.1162	0.179722	newly 5 weeks spec	8949.	5432.52	{4824., 5808.}	40.0898	0.223622	newly 5 weeks cumul	32852.	21052.1	{16785., 24877.}	36.028	0.199505	newly 61 weeks cumul	50701.	35002.	{32552., 35332.}	34.0401	0.176664
18	newly 3 weeks spec	7109.	5666.24	{4910., 6314.}	20.2948	0.100428	newly 3 weeks cumul	17024.	14525.5	{12562., 16478.}	14.7999	0.0719422	newly 5 weeks spec	7086.	4079.75	{3712., 4452.}	42.4252	0.240833	newly 5 weeks cumul	31968.	23830.1	{21466., 25833.}	25.4564	0.128699	newly 59 weeks cumul	50495.	31094.2	{29710., 32109.}	38.4212	0.210788
19	newly 3 weeks spec	9821.	9011.58	{8641., 9644.}	8.28633	0.0392338	newly 3 weeks cumul	23696.	22472.8	{20989., 24317.}	6.24523	0.0291358	newly 5 weeks spec	7954.	6246.53	{5976., 6544.}	21.4668	0.10672	newly 5 weeks cumul	41504.	36918.4	{35604., 38933.}	11.0486	0.0521626	newly 59 weeks cumul	56218.	47139.4	{46651., 48470.}	16.149	0.0777049
20	newly 3 weeks spec	8828.	8775.85	{8495., 9078.}	2.58915	0.0129557	newly 3 weeks cumul	20393.	19468.	{18908., 20241.}	4.66743	0.0225748	newly 5 weeks spec	8356.	10042.5	{9914., 10339.}	21.5728	0.086384	newly 5 weeks cumul	37963.	39623.1	{38842., 40666.}	5.67832	0.025602	newly 57 weeks cumul	56638.	72530.8	{72870., 73634.}	29.4741	0.113984
21	newly 3 weeks spec	9444.	10983.9	{10339., 11244.}	16.3051	0.0654065	newly 3 weeks cumul	18625.	22134.7	{20770., 22734.}	18.8439	0.0747647	newly 5 weeks spec	10527.	12936.9	{12750., 13100.}	22.8927	0.0895002	newly 5 weeks cumul	40402.	48238.9	{46033., 49030.}	19.3972	0.0769028	newly 61 weeks cumul	60507.	90560.2	{88114., 91098.}	49.6689	0.175107
22	newly 3 weeks spec	7683.	5383.44	{3928., 5891.}	29.9305	0.160335	newly 3 weeks cumul	14543.	11142.1	{8400., 12011.}	23.3986	0.121457	newly 5 weeks spec	9422.	8360.95	{5097., 9495.}	11.6902	0.0658768	newly 5 weeks cumul	33493.	26693.6	{19760., 29363.}	20.301	0.105286	newly 59 weeks cumul	53168.	73893.5	{33160., 90205.}	56.6857	0.209065
23	newly 3 weeks spec	16661.	12231.9	{10753., 13524.}	26.5984	0.1363	newly 3 weeks cumul	28675.	21304.	{18806., 23760.}	25.8188	0.131829	newly 5 weeks spec	10882.	14828.9	{14351., 15499.}	36.2783	0.133733	newly 5 weeks cumul	56693.	52221.5	{49248., 55102.}	7.93151	0.0366874	newly 63 weeks cumul	64940.	78369.	{77282., 83688.}	21.3964	0.0838043
24	newly 3 weeks spec	11463.	8749.46	{6861., 10180.}	25.5553	0.131469	newly 3 weeks cumul	20345.	15903.7	{12121., 18776.}	23.95	0.121884	newly 5 weeks spec	12907.	11172.2	{10163., 12000.}	13.6765	0.0655844	newly 5 weeks cumul	47801.	38630.2	{33659., 42434.}	19.6	0.0967603	newly 64 weeks cumul	66552.	58332.5	{54656., 60083.}	12.5405	0.0589318
25	newly 3 weeks spec	11140.	11878.	{8605., 15142.}	18.3613	0.084201	newly 3 weeks cumul	19297.	21317.7	{15237., 28254.}	22.9646	0.10125	newly 5 weeks spec	11133.	9787.8	{7099., 12504.}	18.3546	0.0959768	newly 5 weeks cumul	44204.	44250.1	{40031., 47739.}	7.8639	0.0362889	newly 64 weeks cumul	57475.	55086.	{49316., 65217.}	9.2807	0.0411984
26	newly 3 weeks spec	9973.	6131.27	{5365., 6938.}	38.5213	0.213759	newly 3 weeks cumul	21090.	15314.1	{12860., 17591.}	27.3869	0.141982	newly 5 weeks spec	10139.	4486.65	{4048., 4900.}	55.7486	0.356825	newly 5 weeks cumul	42295.	25502.1	{23013., 28151.}	39.7042	0.221382	newly 58 weeks cumul	65347.	33184.3	{31607., 34203.}	49.2184	0.295383
27	newly 3 weeks spec	9435.	9582.59	{8628., 10774.}	7.72581	0.0333152	newly 3 weeks cumul	19821.	20539.5	{18552., 22629.}	7.55794	0.0319506	newly 5 weeks spec	9500.	9142.34	{7901., 12398.}	17.0371	0.0745885	newly 5 weeks cumul	40049.	40104.4	{36484., 47190.}	8.38618	0.0361453	newly 59 weeks cumul	58495.	59387.2	{51042., 84736.}	18.0276	0.0734525
28	newly 3 weeks spec	6339.	5003.48	{4440., 5495.}	21.1699	0.104975	newly 3 weeks cumul	16315.	14129.5	{12369., 15662.}	13.7909	0.0659237	newly 5 weeks spec	5894.	3523.29	{3271., 3796.}	40.2224	0.224833	newly 5 weeks cumul	28643.	22090.2	{19908., 24010.}	22.9302	0.1143	newly 58 weeks cumul	47081.	29680.9	{28073., 30905.}	36.9577	0.201033
29	newly 3 weeks spec	9036.	9906.99	{8875., 10517.}	11.0878	0.0457107	newly 3 weeks cumul	21261.	22687.	{21024., 23812.}	7.91426	0.0330793	newly 5 weeks spec	8506.	9592.56	{6906., 10874.}	22.2833	0.0922226	newly 5 weeks cumul	39366.	42821.	{36684., 45719.}	12.2114	0.0506538	newly 59 weeks cumul	60373.	69918.1	{48295., 83315.}	25.4772	0.101998
30	newly 3 weeks spec	6346.	6722.69	{5851., 7465.}	10.7115	0.0454225	newly 3 weeks cumul	15783.	16173.4	{14897., 17582.}	6.89292	0.0302175	newly 5 weeks spec	6273.	6776.38	{4088., 8227.}	19.3171	0.0828333	newly 5 weeks cumul	28864.	30134.5	{24757., 33657.}	10.9148	0.0470108	newly 55 weeks cumul	48852.	51451.9	{31443., 63164.}	19.3342	0.0835229
31	newly 3 weeks spec	15806.	14209.4	{11363., 16703.}	13.0726	0.0699499	newly 3 weeks cumul	27617.	26131.7	{18859., 33712.}	18.1252	0.0924624	newly 5 weeks spec	9080.	7562.53	{3947., 11028.}	28.2146	0.154425	newly 5 weeks cumul	52596.	46532.3	{42406., 55441.}	13.7209	0.065646	newly 64 weeks cumul	59587.	52541.9	{48335., 65293.}	14.9121	0.0697685
32	newly 3 weeks spec	10271.	9842.23	{8527., 11094.}	9.52906	0.04214	newly 3 weeks cumul	19060.	18477.3	{15979., 20570.}	10.0776	0.0433536	newly 5 weeks spec	11191.	11144.5	{9836., 12971.}	10.2488	0.0489915	newly 5 weeks cumul	42799.	41713.3	{38267., 45316.}	5.94068	0.0265062	newly 62 weeks cumul	60379.	61148.	{53983., 69373.}	8.09465	0.034632
33	newly 3 weeks spec	12313.	11316.4	{10240., 12093.}	8.36271	0.0387231	newly 3 weeks cumul	26701.	24565.6	{22376., 26793.}	8.50335	0.0393779	newly 5 weeks spec	10094.	8384.24	{7860., 8885.}	17.275	0.0829425	newly 5 weeks cumul	49375.	44055.8	{40628., 46171.}	10.8455	0.0503979	newly 62 weeks cumul	64967.	55875.6	{52856., 57277.}	14.1066	0.0663843
34	newly 3 weeks spec	7987.	5774.37	{5065., 6433.}	27.7029	0.142711	newly 3 weeks cumul	19140.	15828.1	{13155., 18055.}	17.5903	0.0868081	newly 5 weeks spec	7767.	3584.12	{3161., 4050.}	53.8545	0.33753	newly 5 weeks cumul	35440.	24318.7	{21786., 26606.}	31.3806	0.164825	newly 54 weeks cumul	57950.	30175.4	{28586., 31634.}	47.9285	0.28373
35	newly 3 weeks spec	12123.	9445.86	{8649., 12176.}	26.8163	0.13903	newly 3 weeks cumul	20957.	16397.6	{14687., 18069.}	28.365	0.147787	newly 5 weeks spec	11360.	13996.2	{9458., 15258.}	33.1864	0.136603	newly 5 weeks cumul	47195.	43606.1	{42679., 45339.}	7.65763	0.036205	newly 63 weeks cumul	60555.	84250.1	{48891., 91648.}	45.6773	0.164567
36	newly 3 weeks spec	8896.	7485.88	{6202., 8928.}	16.9375	0.0846219	newly 3 weeks cumul	16239.	14338.1	{11835., 16915.}	14.2563	0.0692952	newly 5 weeks spec	12338.	9758.42	{9000., 10399.}	20.9076	0.104409	newly 5 weeks cumul	40963.	33853.3	{29862., 38313.}	17.4627	0.0864429	newly 61 weeks cumul	64577.	54001.7	{52746., 55937.}	16.3763	0.0792269
37	newly 3 weeks spec	11908.	14045.2	{11135., 15711.}	25.1064	0.104907	newly 3 weeks cumul	22407.	28609.7	{17305., 35425.}	39.2847	0.156308	newly 5 weeks spec	9251.	5474.79	{3159., 9308.}	46.082	0.28781	newly 5 weeks cumul	44615.	44700.7	{40871., 47255.}	5.19159	0.0236823	newly 63 weeks cumul	54448.	48278.8	{47040., 49257.}	11.3305	0.0523094
38	newly 3 weeks spec	11993.	12161.7	{11736., 12613.}	2.69165	0.0115731	newly 3 weeks cumul	24507.	24237.	{23367., 25166.}	2.56327	0.011261	newly 5 weeks spec	11210.	13052.2	{11369., 13677.}	18.2012	0.0726762	newly 5 weeks cumul	48530.	51438.2	{48456., 52924.}	6.81014	0.0286254	newly 61 weeks cumul	66756.	83275.4	{67526., 87499.}	25.8487	0.098597
39	newly 3 weeks spec	6786.	5264.73	{4467., 6033.}	22.4178	0.113251	newly 3 weeks cumul	16864.	14730.4	{12043., 17521.}	14.5937	0.0710527	newly 5 weeks spec	6041.	3423.84	{3039., 3787.}	43.3233	0.248399	newly 5 weeks cumul	29945.	22680.3	{19868., 25234.}	24.26	0.122595	newly 58 weeks cumul	46740.	28920.3	{26992., 30780.}	38.1251	0.20902
40	newly 3 weeks spec	7110.	5948.94	{5125., 6600.}	16.539	0.0803683	newly 3 weeks cumul	16261.	14598.3	{12008., 16560.}	11.715	0.0560247	newly 5 weeks spec	7170.	4606.04	{4208., 5014.}	35.7596	0.19326	newly 5 weeks cumul	31380.	24931.3	{21924., 27178.}	20.5504	0.10115	newly 61 weeks cumul	47800.	32983.3	{31411., 34450.}	30.9974	0.161387
41	newly 3 weeks spec	12763.	13758.6	{12060., 15114.}	13.2959	0.0615701	newly 3 weeks cumul	25803.	31073.3	{23974., 37321.}	27.2556	0.113738	newly 5 weeks spec	9905.	4148.9	{2379., 6419.}	58.7398	0.414837	newly 5 weeks cumul	48570.	44231.3	{41421., 47028.}	8.93288	0.0429151	newly 63 weeks cumul	62184.	46845.	{46105., 48536.}	24.6671	0.123659
42	newly 3 weeks spec	7600.	8262.87	{8033., 8458.}	8.72197	0.0362325	newly 3 weeks cumul	17610.	18861.8	{18453., 19285.}	7.10823	0.0297587	newly 5 weeks spec	7814.	9608.6	{9394., 9818.}	22.9665	0.0896818	newly 5 weeks cumul	33674.	37946.9	{37305., 38624.}	12.6891	0.0518402	newly 56 weeks cumul	56523.	75747.6	{75692., 76529.}	34.012	0.126955
43	newly 3 weeks spec	5514.	5458.86	{4812., 5933.}	6.10011	0.0271286	newly 3 weeks cumul	14068.	13794.	{12574., 14816.}	4.81142	0.0214508	newly 5 weeks spec	5382.	5461.03	{4303., 6056.}	10.2934	0.0454825	newly 5 weeks cumul	25003.	25007.3	{22273., 27013.}	5.85914	0.0258652	newly 55 weeks cumul	42797.	43523.7	{34410., 47860.}	10.1175	0.0444926
44	newly 3 weeks spec	9897.	9212.19	{7651., 10476.}	9.53612	0.0451087	newly 3 weeks cumul	17922.	17096.6	{14173., 19292.}	9.10462	0.0422884	newly 5 weeks spec	12427.	11828.9	{9984., 12967.}	8.19297	0.0379198	newly 5 weeks cumul	43675.	40994.8	{35137., 45447.}	8.12698	0.0378969	newly 62 weeks cumul	65197.	64097.	{54980., 69534.}	9.18421	0.0409358
45	newly 3 weeks spec	7988.	5744.06	{4648., 6601.}	28.1838	0.148144	newly 3 weeks cumul	17198.	13599.9	{10690., 16258.}	21.211	0.108572	newly 5 weeks spec	9497.	4935.43	{4221., 5358.}	48.0317	0.288759	newly 5 weeks cumul	36455.	24370.1	{21009., 26552.}	33.1501	0.177915	newly 60 weeks cumul	60722.	33780.7	{31878., 34517.}	44.3683	0.256737
46	newly 3 weeks spec	8753.	8376.75	{7145., 9647.}	8.62916	0.0399232	newly 3 weeks cumul	17795.	17095.8	{14791., 19210.}	8.44046	0.0388098	newly 5 weeks spec	10647.	8908.75	{8470., 9468.}	16.3262	0.0789066	newly 5 weeks cumul	39229.	35793.3	{32542., 39354.}	9.15861	0.0432332	newly 59 weeks cumul	63639.	52569.4	{51371., 54336.}	17.3943	0.0838818
47	newly 3 weeks spec	6293.	4411.68	{3865., 4912.}	29.8954	0.155914	newly 3 weeks cumul	16455.	13474.9	{11910., 14805.}	18.1183	0.0882928	newly 5 weeks spec	6515.	2827.83	{2394., 3197.}	56.5951	0.364901	newly 5 weeks cumul	29490.	20018.	{18024., 21671.}	32.1195	0.169401	newly 55 weeks cumul	54744.	26034.3	{23515., 27628.}	52.4435	0.323667
48	newly 3 weeks spec	8060.	6266.25	{5081., 7750.}	24.3803	0.124269	newly 3 weeks cumul	18107.	15819.5	{12625., 19380.}	15.9797	0.077899	newly 5 weeks spec	8793.	4538.6	{3773., 6962.}	48.3839	0.296524	newly 5 weeks cumul	35919.	26173.5	{21878., 33508.}	27.6875	0.14481	newly 60 weeks cumul	61312.	33938.1	{30537., 48269.}	44.6469	0.261585
49	newly 3 weeks spec	13841.	12962.6	{8268., 16395.}	17.7383	0.0943901	newly 3 weeks cumul	23044.	22145.6	{12093., 31487.}	24.6452	0.126485	newly 5 weeks spec	12770.	9901.74	{4775., 17148.}	38.5106	0.21823	newly 5 weeks cumul	52454.	45991.5	{39101., 52742.}	13.3849	0.0653604	newly 64 weeks cumul	65680.	58739.	{48579., 92673.}	27.6261	0.126261
50	newly 3 weeks spec	12684.	13377.7	{12166., 14447.}	7.47714	0.0312266	newly 3 weeks cumul	25589.	27847.6	{23747., 31864.}	12.5823	0.051211	newly 5 weeks spec	9733.	6993.15	{5209., 8871.}	28.3932	0.156071	newly 5 weeks cumul	48457.	46312.7	{44023., 48850.}	5.33165	0.0239657	newly 63 weeks cumul	60105.	52627.2	{50044., 58905.}	12.9527	0.0607933
